# Materials-driven strategies in bacterial engineering

**DOI:** 10.1557/s43579-024-00623-7

**Published:** 2024-07-31

**Authors:** Giuseppe Maria Paternò

**Affiliations:** Physics Department, Politecnico Di Milano, Piazza L. da Vinci 32, 20133 Milano, Italy; Center for Nanoscience and Technology, Istituto Italiano Di Tecnologia, Via Rubattino 71, 20134 Milano, Italy

## Abstract

This perspective article focuses on the innovative field of materials-based bacterial engineering, highlighting interdisciplinary research that employs material science to study, augment, and exploit the attributes of living bacteria. By utilizing exogenous abiotic material interfaces, researchers can engineer bacteria to perform new functions, such as enhanced bioelectric capabilities and improved photosynthetic efficiency. Additionally, materials can modulate bacterial communities and transform bacteria into biohybrid microrobots, offering promising solutions for sustainable energy production, environmental remediation, and medical applications. Finally, the perspective discusses a general paradigm for engineering bacteria through the materials-driven modulation of their transmembrane potential. This parameter regulates their ion channel activity and ultimately their bioenergetics, suggesting that controlling it could allow scientists to hack the bioelectric language bacteria use for communication, task execution, and environmental response.

## Introduction

Bacteria play a pivotal role in diverse ecosystems, ranging from soil and oceans to the Earth’s atmosphere and, critically, the human body. As essential drivers of nutrient cycling, symbiotic relationships, and ecological balance, bacteria profoundly influence the health and sustainability of various environments. From the production of oxygen through photosynthesis to the colonization of diverse niches within the human body, including the gastrointestinal tract,^[1]^ epidermis, and even the once-thought-aseptic environment of the brain,^[2]^ bacteria have played a profound role in the genesis and evolution of humans. Such continuous interaction of bacteria with diverse environments, as well as their strong adaptability, have conferred them with unique attributes finely tuned by evolution. Here I list some relevant examples, alongside with the related applications:

- Photosynthetic bacteria harness the power of sunlight to produce energy, a process with applications in sustainable energy production, such as bio-solar cells and artificial photosynthesis^[3]^;
- Electrogenic bacteria possess the ability to promote redox reactions through extracellular electron transfer pathways, offering potential applications in microbial fuel cells and electrochemical cells for electricity generation and environmental remediation;
- Bacteria can exhibit motility,^[4, 5]^ allowing them to navigate diverse environments. This feature has important implications for micromotor technology^[6, 7]^ and targeted drug delivery systems in body locations that are difficult to reach or navigate due to the low Reynold’s number,^[8]^ *i.e*., the gastrointestinal tract or tumors^[9, 10]^;
- Bacteria can assemble into microbial communities with sophisticated decision-making processes,^[11–13]^ influencing the health of crops and humans and offering opportunities for the production of engineered living materials with tailored properties.

In the last decades, the relatively new field of synthetic biology seeks to exploit the unique properties of these microorganisms for transformative applications.^[14]^ This represents a state-of-the-art technique for serving to this purpose.^[15, 16]^ By rewiring genetic circuits and metabolic pathways, synthetic biologists can tailor bacteria to extend and augment their living attributes, such as their ability to harvest light energy^[17]^ or undergo extracellular electron transfer,^[18]^ as well as to encode microbes with programmable functionalities, such as the responsivity to optical and chemical stimuli.^[19]^ While a significant portion of synthetic biology focuses on the engineering of genetic components, it is important to acknowledge that material approaches can also have profound effects on bacterial gene expression and protein function. The integration of abiotic materials with bacterial systems can influence genetic pathways in ways that complement traditional synthetic biology techniques. For instance, recent developments have demonstrated the potential of electronically controlling gene expression using materials-based systems.^[20]^

Focusing on materials as a “stand-alone” tool for controlling the fate of eukaryotic cells and organisms, our research community has recently turned its attention to the use of exogenous materials for this purpose. The general approach involves utilizing exogenous nano/molecular materials: their nanometer-scale dimensionality is essential for establishing reliable abiotic interfaces with biological molecules and cells. Additionally, the artificial functionalities of these materials, such as optical and electronic responsiveness, enable the equipping of living matter with extra mechanisms that complement existing ones.^[21–23]^ This relatively new paradigm has now extended into microbes.^[14]^

**Figure 1:**
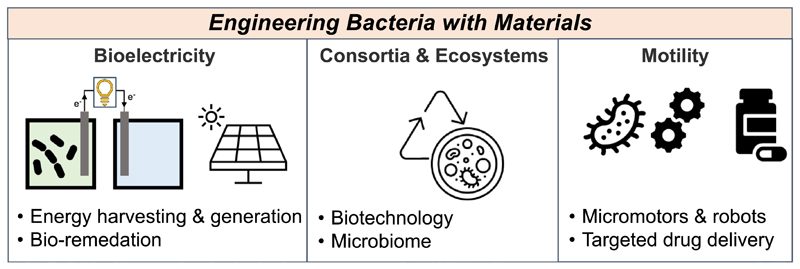
Schematic summarizing the general contents in this perspective.

In the past, the interaction between materials and microbes has been extensively studied and exploited for bacterial eradication and the treatment of related infections,^[24–26]^ as well as for the development of bacterial sensors to hinder the spread of pathogenic bacteria.^[27–32]^ However, the current approach involves viewing bacteria as engineerable materials, with the goal of achieving new functionalities or enhancing existing ones through material interfaces,^[33]^ for instance, by injecting energy to bacteria to allow them to operate away from equilibrium.^[34]^ Beyond investigating the fascinating world of bacterial electrophysiology and complex communication,^[13, 35, 36]^ this approach can offer unprecedented opportunities to address pressing societal challenges. These include the development of new and intrinsically sustainable energy harvesting systems based on bio-mimetic photosynthetic hybrids,^[37–40]^ microbial fuel cells for power production,^[41]^ bio-electrosynthesis platforms where microbes produce desired chemicals,^[42]^ and novel engineered living systems that combine features of materials with living attributes that have been refined by evolution, such as motility and adaptation (Fig. 1).^[43–45]^

In this perspective article, I explore the multifaceted landscape of material-driven strategies in bacterial engineering. For clarity, in this article, I use this term to refer to the fine manipulation of bacterial functions driven by the interface with exogenous abiotic materials, with the general aim of augmenting existing features or conferring new ones. Additionally, note that the scope of this manuscript is not to provide a comprehensive overview of the extensive literature on microbial engineering. Instead, I aim to highlight recent interdisciplinary research efforts that share common materials-driven approaches, serving the purpose of bacterial engineering. For each specific topic I will show only a few recent case studies that are summarized in Table I. First, I will give a brief overview on the use of carbon-based materials for the enhancement of bioelectric capabilities of bacteria to undergo extracellular electron transfer (Section “Introduction”), as well as to enhance the ability of photosynthetic microbes to harvest sunlight (Section “Engineering bioelectricity generation through material interfaces”). In Section “Advancements in photosynthetic biohybrid systems”, I will present recent studies on the use of functional materials to modulate bacterial communities, while in the Section “Microbial communities and materials” I will briefly talk about the fascinating opportunities arising from the use of bacteria as biohybrid microrobots. In Section “Conclusion”, I will discuss a general paradigm for engineering bacteria, consisting of the materials-driven modulation of their transmembrane potential. This is essentially the parameter that regulates their ion channel activity and ultimately their bioenergetics, implying that achieving control over it would permit hacking of the bioelectric language that bacteria use for communicating, carrying out tasks, and responding to their environment.^[46]^

### Engineering bioelectricity generation through material interfaces

The interaction between materials and bacteria has garnered significant attention for its potential to engineer bioelectricity generation. The general rationale is to exploit and possibly enhance the mechanism that certain bacteria have developed to transport electrons extracellularly as part of their respiration. This ability can be then applied to develop microbial bioelectronic devices, such as microbial fuel cells (MFCs) and microbial electrolysis cells (MECs). In MFCs, microbes oxidize organic substrates and transfer respiratory electrons to the anode,^[47]^ while in MECs, electrons are injected into microbes from the cathode, which in turns utilize the electrons as reducing equivalents for their metabolism and generate desired chemicals.^[48]^ In either way, material abiotic interfaces, particularly those designed with conductive or semiconductive properties, can facilitate such electron transfer, thus enhancing the natural bioelectric capabilities of microbial communities. The material/microbe interface can be usually engineered at the electrode level,^[49]^ or at the microbe/microbe interface, *i.e*., to improve charge conduction and thereby collection at the electrode. From the bacterial side, I will focus on *Shewanella oneidensis* (strain MR-1). Although the most studied electrochemically active bacteria are *She-wanella oneidensis* and *Geobacter sulfurreducens*, the former strain has gained more popularity as model organism, because it is a facultative anaerobe allowing ease of cultivation and due to its annotated genome sequence that ensures facile genetic manipulation.^[50, 51]^ From the materials side, here I will show two relevant examples of the utilization of carbon-based materials as abiotic systems, which are in general more biocompatible and bio-mimetic than their inorganic counterparts, supporting both electronic and ionic conduction.^[52]^

**Figure 2:**
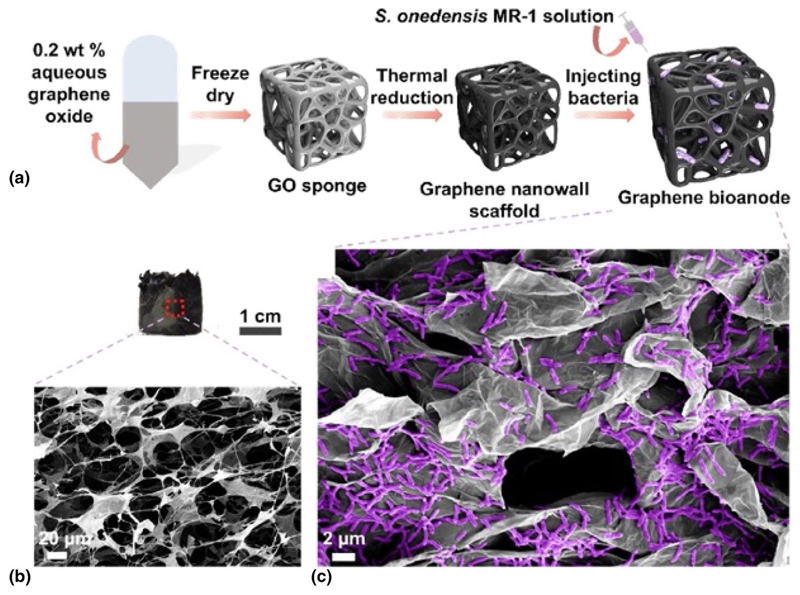
(a) Schematic illustration of the bioengineering steps. (b) Optical image of the graphene electrode (upper) and SEM image of its porous sponge morphology (below). (c) SEM image of the graphene-based bioanode that hosts a living population of *S. oneidensis* after electrochemical testing for 8 days. Reproduced with permission from Ref. 53. Copyright 2023, Elsevier.

Regarding the engineering of the electrode, in a recent article Andreeva, Novoselov, and collaborators have developed a novel biocompatible anode for MFCs based on graphene (Fig. 2).^[53]^ The relatively high electrical conductivity and low dimensionality of graphene can be advantageous for the development of novel lightweight bioanodes for new-generation energy technologies; however, its relatively low hydrophilicity is not compatible with the exposure to biological environments. In this study, the authors have shown that the conductive graphene nanowalls form biocompatible and hydrophilic micro-confinements that effectively concentrate the biomass density of electrogenic *Shewanella oneidensis*. This graphene-based bioanode demonstrates a stable and rapid response, achieving a steady-state bio-current density of 135.35 mA m^−2^ within just a few hours.

**Figure 3:**
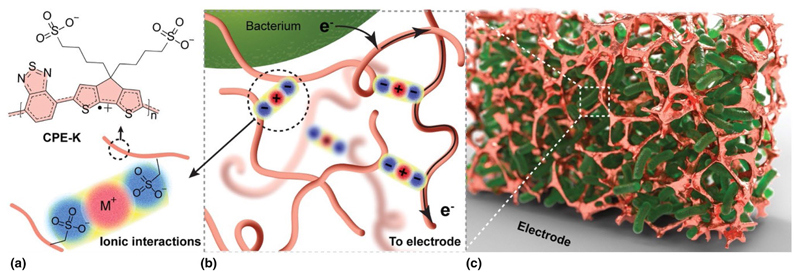
Bioelectronic composite concept and initial biocompatibility testing. (a) The molecular structure of CPE-K is shown with a polaron (radical cation) in its backbone. CPE-K is expected to form a hydrogel via ionic interactions. (b) Metabolic electrons are transferred extracellularly to the matrix made of conductive CPE-K polymer chains. (c) A cartoon representation of a conductive matrix (metallic color) encapsulating electroactive bacteria (green). Reproduced with permission from Ref. 42. Copyright 2020, Wiley & Sons.

Enhancement of bioelectricity extraction can be also achieved through the utilization of functional material interfaces that are able to establish efficient percolation pathways for the bio-generated charges. Within this context, McCuskey et al. have reported on the use of conjugated polyelectrolyte CPE-K, which works as a conductive matrix to electronically connect a three-dimensional network of *Shewanella oneidensis* to a gold electrode, hence increasing bio-current ≈150-fold over control biofilms (Fig. 3).^[42]^ These bio-composites spontaneously assemble from solution, forming an intricate arrangement of cells within a conductive polymer matrix. This assembly not only increases the bio-current due to a higher number of cells interfacing with the electrode but also enhances the current extracted per cell, indicating efficient long-range electron transport. Additionally, the bio-composites exhibit nearly an order-of-magnitude lower charge transfer resistance compared to CPE-K alone. This supports the notion that the electroactive bacteria and the conjugated polyelectrolyte synergistically contribute to creating an effective bioelectronic composite.

### Advancements in photosynthetic biohybrid systems

Photosynthetic organisms have developed a complex machinery, consisting of pigment–protein complexes and molecules, to capture sunlight and store it as chemical energy. The exploitation of such evolutionary ability of photosynthetic microbes represents one of the most fascinating routes to produce clean and renewable energy.^[54]^ To this end, coupling photosynthetic bacteria with nano/molecular materials have turned to be a successful strategy to enhance this useful microbial capability and develop biohybrid photovoltaic and photocatalytic cells.^[55]^ The relatively small dimension of such systems is well suited for establishing diffuse abiotic interfaces with bacteria, which are ≈ 10 × smaller than eukaryotic cells. Here, I will give three relatively recent examples of how biocompatible molecular and nanostructured semiconductors can be used to serve to this role.

Boghossian and co-workers have reported on the double purposes use of single-walled carbon nanotubes (SWCNTs) as functional interfaces for the photosynthetic bacterium *Synechocystis*.^[56]^ First, the authors showed that bacterial cells uptake efficiently SWCNTs, which interestingly are also inherited by daughter cells. The near-infrared emission of the homogeneously distributed SWCNTs allows for imaging of *Synechocystis*, without any spectral overlap with the relatively strong autofluorescence of cyanobacteria in the visible spectrum. Furthermore, these hybrid living cells retained photosynthetic activity and showed an improved photo-exoelectrogenicity when incorporated into bioelectrochemical devices.

**Figure 4:**
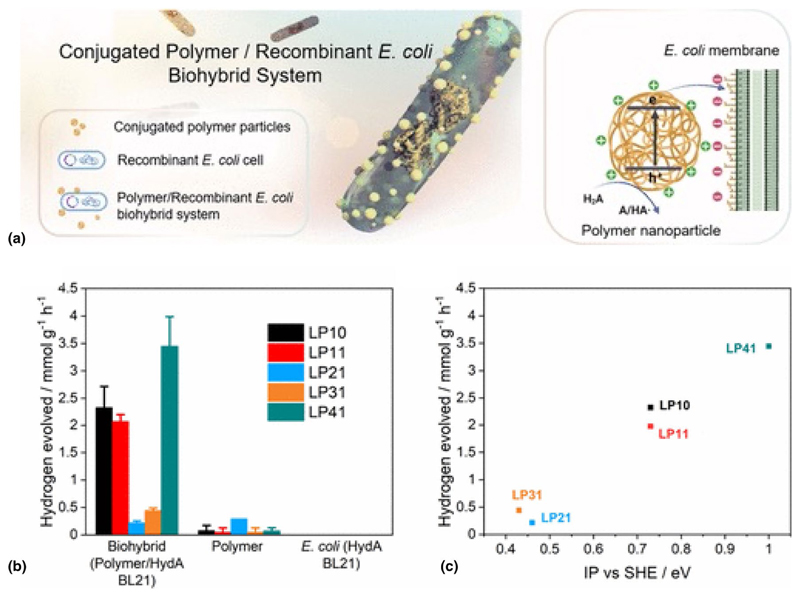
(a) Strategy for the assembly of the conjugated polymer particle/recombinant *E. coli* biohybrid system mainly based on electrostatic interactions with one-hole (HA·/H2A) oxidation of ascorbic acid for hydrogen formation. (b) Hydrogen production performance of biohybrid systems (4.3-mL 50-mg L^−1^ polymer nanoparticle solution, 200 μL of *E. coli* with OD600 ≈ 1.0) compared to the polymer nano-particle and *E. coli* HydA BL21 control groups after 3 h of irradiation. (c) Correlation between the biohybrids’ hydrogen evolution activity and the polymer IP values. Plots and error bars represent the averages and standard deviations of at least two assays. All values were normalized to the polymer amount, and all measurements were conducted in 10-mM Tris–HCl buffer under irradiation of an AM 1.5G solar simulator. Adapted from Ref. 57. This publication is licensed under CC-BY 4.0.

In a recent study, Ying Yang et al. have shown that the photobiocatalytic activity of *E. coli* can be enhanced via the use of conjugated polymers (Fig. 4).^[57]^ Organic semiconductors present several advantages for applications in bioelectronics, such as their synthetically tunable optoelectronic properties and surface properties that are derived from a wide range of accessible monomers and supramolecular assembly patterns,^[58]^ their biocompatibility, and, as I have already stated, the possibility to support ionic and electronic conduction. The authors showed that under simulated solar light irradiation, the biohybrid system comprising the fluorene/dibenzo[b,d]thiophene sulfone copolymer (LP41) and recombinant *E. coli* exhibits a sacrificial hydrogen evolution rate of 3.442 mmol g^−1^ h^−1^. This rate is more than 30 times higher than that of the polymer photocatalyst alone, which produces 0.105 mmol g^−1^ h^−1^. In contrast, no detectable hydrogen is generated by the *E. coli* cells alone.

Another successful approach to increase the efficiency of the photosynthetic process passes through refinement and engineering of the electrode/bacteria interface. In these regards, Zhang et al. have developed an aerosol jet printing method for generating hierarchical electrode structures using indium tin oxide nanoparticles.^[59]^ The authors demonstrated that when integrated with the cyanobacterium *Synechocystis*, micropillar array electrodes featuring microbranches demonstrated superior biocatalyst loading, enhanced light utilization, and increased electron flux output. This configuration nearly doubled the photocurrent compared to state-of-the-art porous structures of the same height. By increasing the micropillars’ heights to 600 µm, the system achieved milestone mediated photocurrent densities of 245 µA cm,^[2]^ approaching theoretical predictions—and external quantum efficiencies of up to 29%.

### Microbial communities & materials

The interaction of microbial communities with their host environments is foundational to human evolution and the maintenance of diverse ecological cycles. Bacteria can prosper in virtually any ecological niche because they possess sophisticated machineries that operate at the community level, allowing them to collectively respond to external stressors and adapt to their surroundings. In this context, materials can be used to modulate such cooperative behavior, with the goal of offering new and unprecedented tools that may serve both fundamental and applied research purposes. In this regard, the palette of materials employed is rather wide, spanning from inorganic systems, such as minerals and metals, to organic systems and hydrogels, with the aim of mimicking and capturing the complexity of hybrid natural materials (i.e., soil). Here, I will show three examples on the exploitation of the dynamical microbe–material interaction for building up artificial platforms, with the aim to modulate microbial consortia functionalities and perhaps assemble new hybrid ecosystems.^[60]^

Tian and collaborators have recently reported a soil-inspired chemical system that consists of nanostructured minerals, starch granules, and liquid metals.^[61]^ The authors demonstrated that such a composite, which was fully characterized via optical and structural techniques, is able to enhance biofilm growth, bacterial growth and biofuel production. Interestingly, in vivo studies demonstrate that the material enhances gut bacterial diversity and corrects bacterial dysbiosis under pathophysiological conditions. Additionally, it effectively protects the gastrointestinal epithelium and alleviates colitis symptoms in a rodent model of dextran sulfate sodium (DSS)-induced colitis. Looking ahead, this chemical system has potential applications beyond the gut microbiota, extending to the study of skin and soil microbiomes.

Wang et al. have reported a new concept of engineered living material, in which they made use of biofilm-mediated mineralization to construct structurally ordered, environmentally adaptive composite materials.^[44]^ In particular, the authors demonstrated light-inducible bacterial biofilm formation coupled with bio-mimetic hydroxyapatite (HA) mineralization. By modulating the growth of functional biofilms, they could precisely control both the location and degree of mineralization, achieving spatial and biomass density regulation. The embedded cells remained viable, capable of sensing and responding to environmental cues. Post-mineralization, the composites exhibited up to a 15-fold increase in Young’s modulus, making them suitable for spatially controlled damage repair. This study not only offers insights into the formation mechanisms of natural graded composites but also presents a practical approach to creating living composites with dynamic responsiveness and environmental adaptability.

**Figure 5:**
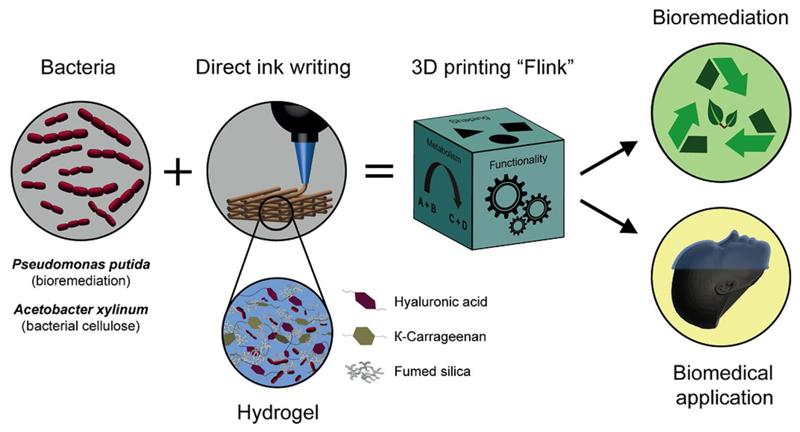
The diagram illustrates a 3D bacteria-printing platform designed for creating functional living materials. This method embeds multifunctional bacteria in a bioink composed of biocompatible hydroxyapatite (HA), κ-carrageenan (κ-CA), and food starch (FS) within a bacterial medium. The process enables 3D printing of bacteria-containing hydrogels into arbitrary shapes, leveraging the diverse products of bacterial metabolism for added functionality. Incorporating specific bacterial strains results in a living, responsive hydrogel, termed Flink. For instance, including *P. putida* allows the material to degrade environmental pollutants, while *A. xylinum* enables the formation of bacterial cellulose in situ, offering potential for biomedical applications. Reproduced from Ref. 62. This publication is licensed under CC-BY 4.0.

Schaffner et al. have exploited the intrinsic capability of bacteria to dynamically respond in a cooperative way to the surrounding environment to develop new functional materials, which consist of a bacterial biofilm embedded in a biocompatible and functionalized 3D printing ink. In this way, the authors could print two kinds of living materials, which are capable of degrading pollutants and of producing medically relevant bacterial cellulose.^[62]^ This innovative bacteria-printing platform enables the creation of complex materials with precise spatial compositions, geometries, and properties that traditional technologies cannot achieve, opening up new possibilities for biotechnological and biomedical applications (Fig. 5).

### Materials-driven engineering of motile *bacteria* as microrobots

Bacteria exhibit a diverse array of motility mechanisms that allow them to navigate and exploit various environments.^[63]^ These evolutionary traits, shared with other organisms,^[64–68]^ enable bacteria to manipulate objects at the micro/nanoscale and perform tasks, such as drug delivery. The operation of such micro-biobots can occur even in challenging biological settings with low Reynolds numbers or hard-to-reach body locations and tumors.[^6, 69–72]^ The general rationale here is to confer tacticity to bacteria by interfacing them with responsive materials. Remote control of such biological robots is, of course, required; thus, the materials must respond on demand to remotely triggered stimuli, such as light pulses or magnetic field.

**Figure 6:**
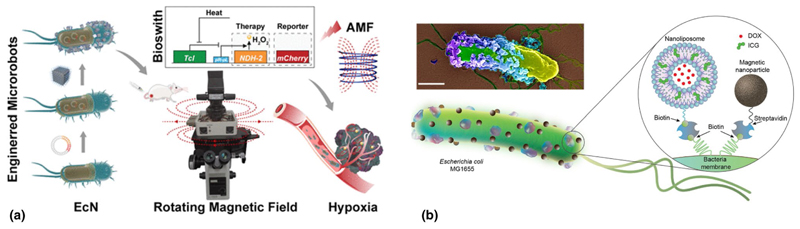
(a) Construction, characterization of engineered bacteria hybrid microrobots, and application for cancer treatment. Adapted from Ref. 73. Copyright 2022, American Chemical Society. (b) Schematic illustration of the bacterial biohybrid microrobots, conjugated with NLs and mNPs. NLs are loaded with DOX and ICG, and both NLs and mNPs are conjugated to bacteria via biotin–streptavidin interactions. Inset shows an SEM image of an example bacterial biohybrid carrying mNPs and NLs. Image was pseudocolored. Scale bar, 500 nm. Adapted from Ref. 74. This publication is licensed under CC-BY 4.0.

Chen et al. reported a biohybrid microrobot integrated with magnetic, thermal, and hypoxia sensitivities, featuring an internal fluorescent protein as a dual reporter of thermal and positioning signals for targeted cancer treatment [Fig. 6(a)].^[73]^ This microrobotic system comprised three key components: magnetic nanoparticle (MNP)-loaded probiotic *Escherichia coli* Nissle1917 for spatial magnetic and hypoxia perception, a thermal logic circuit engineered into the bacteria to control the biosynthesis of mCherry as the temperature and positioning reporter, and the NDH-2 enzyme encoded in the EcN for enhanced anticancer therapy. Utilizing fluorescent-protein-based imaging feedback, the microrobot demonstrated excellent thermal sensitivity and active targeting ability to tumor areas collectively under a magnetic field. The hybrid microrobot efficiently induced cancer cell apoptosis both in vitro and in vivo, aided by magnetothermal ablation and NDH-2-induced reactive oxygen species (ROS) damage. This study highlights the potential of the biohybrid microrobot as an ideal platform that integrates physical, biological, and chemical properties for collective perception and propulsion in targeted cancer treatment.

Sitti and collaborators proposed a method to confer magneto-sensitivity to motile bacteria, aiming to construct magnetically steerable bacterial microrobots for cargo delivery in biological environments [Fig. 6(b)].^[74]^ Magnetic nanoparticles and nanoliposomes, loaded with photothermal agents and chemotherapeutic drugs, were successfully integrated into *Escherichia coli*. These bacterial biohybrids outperformed previously reported *E. coli*-based microrobots by retaining their inherent motility. They navigated through biological matrices and colonized tumor spheroids under magnetic field influence. Upon near-infrared stimulation, these biohybrids could release drug molecules on demand. This work presents a multifunctional microrobotic platform, enabling guided movement within 3D biological environments and stimuli-responsive therapeutic delivery for various medical applications.

### Materials for hacking the bacterial bioelectric language

The objective of engineering living matter is to perturb and potentially modulate biological attributes to enhance and utilize the unique capabilities of living organisms. One common approach involves making living matter responsive to specific stimuli, using either synthetic biology tools^[75]^ or functional materials,^[22, 76]^ to modulate the electrophysiology and activity of cells and organisms. This approach is also applicable to bacteria, even though the relationship between their electrophysiology, bioelectricity, bioenergetics, and behavior has only recently begun to be understood. Recent research has shown that bacterial membrane potential is not a static parameter, but rather a dynamic one that plays a signaling role,^[77–80]^ similar to the Hodgkin & Huxley model for neuronal signaling.^[81]^ Bacteria exhibit a complex bioelectric signaling language that regulates their metabolism, behavior, and function within microbial communities.^[35, 82, 83]^ Since the membrane potential dynamic mediates this language, controlling this parameter could serve as a general and intriguing strategy for engineering bacteria. For these reasons, despite bacterial bioelectricity represents a general features that is relevant virtually to all the sections of this perspective, I prefer adding more emphasis on this topic by presenting it in separate section.

In a seminal study, Kralji et al. observed neuron-like electrical spiking in *E. coli* when exogenous electrical pulses were applied through field stimulation electrodes.^[84]^ This discovery launched the field of bacterial electrical stimulation, demonstrating that bacteria can actively sense their environment via their electrical potential. More recently, Asally and colleagues showed that exogenous electrical bias causes hyperpolarization in unperturbed cells, while pre-exposed cells (to UV light or antibiotics) depolarize under the same stimulation.^[85]^ Additionally, Süel and collaborators found that electrical stimuli can promote the proliferation of motile cells in bacterial consortia.^[86]^ These studies indicate that bacterial processes can be actively manipulated using exogenous stimuli.

Recently, the application of abiotic material interfaces to activate and study cellular bioelectric pathways, initially successful in neuronal stimulation, has been extended to bacteria. The rationale here consists in the use of molecular or nanostructured materials, which can transduce the energy provided by an external stimulus (*i.e*., electrical or optical) into the modification of one or more cell membrane physical parameters, such as its resistance through the opening/closure of ion channels or modulation of membrane permeability and the electrical capacitance.^[76]^ The material must be stable, non-toxic, and capable of localizing within or near the membrane to deliver transient (down to millisecond level), localized, and controllable (in duration and intensity) perturbations to cells.

Gao et al. reported on the use of multiscale, structured silicon materials as non-genetic optical transducers capable of modulating the activities of both single bacterial cells and bio-films with high spatiotemporal resolution.^[87]^ They discovered a novel form of rapid, photothermal gradient-dependent intercellular calcium signaling within biofilms and an unexpected coupling between calcium dynamics and biofilm mechanics, which could be crucial for biofilm resistance.

Payne and collaborators recently demonstrated that gold ions can hyperpolarize bacteria.^[88]^ Using single-cell fluorescence imaging, they observed that gold ions hyperpolarize *B. subtilis* and *E. coli* in a concentration-dependent manner. These ions are generated electrochemically, with their concentration controlled by tuning the voltage and frequency of an external electrical bias. The electrochemically generated gold ions diffuse through the imaging chamber, creating a wave of hyperpolarization whose speed can be modulated by voltage and frequency.

**Figure 7:**
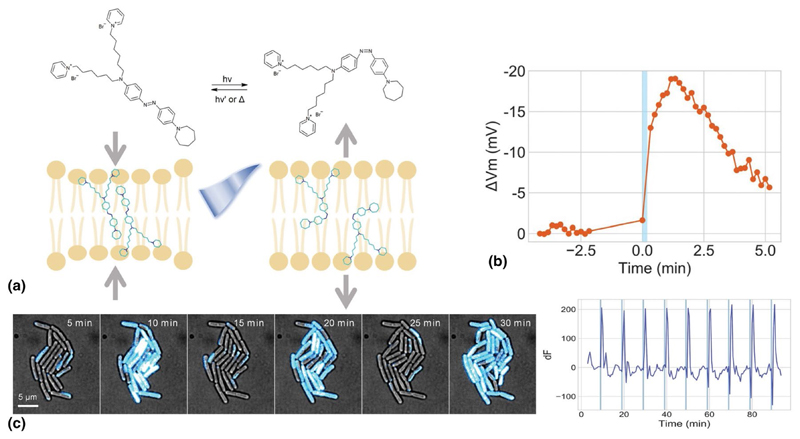
(a) Illustrative diagram depicting the photo-induced isomerization of Ziapin2. This process highlights the optomechanical action of Ziapin2 when integrated into the lipid membrane. In its elongated trans form, Ziapin2 can dimerize within the membrane, resulting in reduced membrane thickness and increased membrane capacitance. Conversely, exposure to cyan light (470 nm) induces isomerization to its bent cis form, which disrupts the dimers. This transition increases membrane thickness and decreases membrane capacitance.^[90–95]^ Representative single-cell time-trace of *Ziapin*-induced membrane potential dynamics before and after 470-nm light stimulation. (c) Periodic photo-induced hyperpolarization. Cells were cultured with Ziapin2 and stimulated by 470-nm light for 10 s every 10 min. Membrane potential was measured using TMRM. The change in TMRM fluorescence (dF) over time from a representative microcolony. Adapted from Ref. 89. This publication is licensed under CC-BY 4.0.

In these regards, my group and I have shown that precise optical modulation of bacterial membrane potential can be achieved through a materials-based approach (Fig. 7).^[89]^ We employed a membrane-targeted azobenzene molecule, *Ziapin2*, to modulate membrane capacitance and potential via an opto-mechanical effect. When exposed to visible light (λ ≈ 470 nm), *Ziapin2* induced a transient hyperpolarization followed by a depolarization rebound, displaying an intriguing oscillatory pattern. The discrepancy between the rapid isomerization process and the prolonged biological effects led us to investigate the involvement of voltage-gated ion channels. Remarkably, we discovered that the potential modulation triggered by *Ziapin2* isomerization activates the chloride channel, whose role in prokaryotes remains largely uncharacterized. These findings suggest that bacteria possess bioelectric machinery capable of responding to rapid voltage changes.

## Conclusion

In conclusion, the dynamic interplay between bacteria and material interfaces opens new avenues for bioengineering, with the potential to revolutionize multiple fields, including energy production, environmental remediation, and medical applications. The general rationale is to couple and harmonize existing biological attributes, which are defined by evolution, with exogenous ad hoc functionalities borrowed from materials. Ultimately, this materials-driven paradigm has the potential to be applied broadly to any bacterial species, whereas genetic rewriting can suffer from a limited range of applicability.

In addition to standard applications of engineered microbes in energy harvesting/storage and environmental remediation, I want to highlight two novel research routes that may stem from this field, namely, i. achieving control over the morphogenesis, composition, and functionality of microbial communities and ecosystems and ii. hacking the bioelectric code of bacteria. The development of the former research field has important implications for the study and engineering of microbiomes, such as the human gut or epidermis microbiomes. The latter would allow us to explore the uncharted territory of bacterial bioelectricity, which, in my view, represents the software that regulates their activity.

## Figures and Tables

**Table 1:** Summary of the case studies reported in this perspective paper, categorized by bacteria, materials, and the resulting functions from the material interfacing.

Bacteria	Materials	Applications/functions
*Shewanella oneidensis*	Graphene Conjugated polyelectrolyte	Microbial fuel/electrochemical cells Improve charge collection Improve charge extraction
*Synechocystis* *E. coli*	Carbon nanotubes Conjugated polymers Indium Tin Oxide NPs	Hybrid photosynthetic systems Enhance photo-exoelectrogenicity Improve photocurrent collection
Gut microbiota bacteria *E. coli* *A. xylinum / B. subtilis*	Artificial soil Hydroxyapatite Hydrogel ink	Modulation of microbial communities Enhancement of gut bacterial diversity Modulation of mineralization Degradation of pollutants
*E. coli*	Magnetic NPs	Bacterial microrobots Cancer cells ablation Drug delivery
*B. subtilis*	Silicon nanowires Gold NPs Azobenzenes	Modulation of bacterial bioelectricity Photomodulation of calcium signaling Electromodulation of cell potential Photomodulation of cell potential

## Data Availability

Any inquiries on the data can be directed to the Corresponding Author.
